# Burden and genotype distribution of high-risk *Human Papillomavirus* infection and cervical cytology abnormalities at selected obstetrics and gynecology clinics of Addis Ababa, Ethiopia

**DOI:** 10.1186/s12885-019-5953-1

**Published:** 2019-08-05

**Authors:** Kirubel Eshetu Ali, Ibrahim Ali Mohammed, Mesfin Nigussie Difabachew, Dawit Solomon Demeke, Tasew Haile, Robert-Jan ten Hove, Tsegaye Hailu Kumssa, Zufan Lakew Woldu, Eshetu Lemma Haile, Kassu Desta Tullu

**Affiliations:** 1 International Clinical Laboratories, Addis Ababa, Ethiopia; 2 Department of Medical Laboratory Sciences, College of Health Sciences, Addis Ababa University, Addis Ababa, Ethiopia; 3 St. Paul Millennium Medical College Hospital, Addis Ababa, Ethiopia; 4 SinamokshEthio Women’s Health Special Clinic, Addis Ababa, Ethiopia; 5 Armauer Hansen Research Institute, Addis Ababa, Ethiopia; 6 Hemen Maternal and Children Health Specialty center, Addis Ababa, Ethiopia

**Keywords:** High-risk *Human Papillomavirus*, Cervical cytology, Obstetrics and gynecology, Genotype distribution, Real-time PCR, Pap cytology

## Abstract

**Background:**

*Human papillomavirus* is recognized as a major cause of cervical cancer. It is estimated that annually, 7,095 women are diagnosed with cervical cancer and 4,732 die from the disease in Ethiopia. Understanding that the screening practice is very poor and the coverage is very limited, this disease burden is one of the major public health agendas in Ethiopia. This study aimed to assess the burden and genotype distribution of high-risk *human papillomavirus* (HR HPV) infection and cervical cytology abnormalities at selected obstetrics and gynecology clinics of Addis Ababa, Ethiopia.

**Methods:**

An institutional-based cross-sectional study design was employed from June to October 2015. Cervical samples were collected from 366 participants based on inclusion criteria. HR HPV DNA was analyzed using an Abbott Real-Time PCR system, and cervical cytology screening was performed using the conventional Pap-smear technique. Data were entered in to Epi-data version 13 and analyzed using STATA version 11.

**Results:**

The overall HR HPV burden and abnormal cytology were 13.7 and 13.1%, respectively. The majority of HR HPV types were other than types 16 and 18. Of the total abnormal cytology results, 81.3% were low-grade squamous intraepithelial lesions (LSILs), and 12.5 and 6.3% were atypical squamous cells of undetermined significance (ASCUS) and high-grade squamous intraepithelial lesions (HSILs), respectively. Residence, occupation, and HIV serostatus were significantly associated with HR HPV infection. Among the variables, age, age at first marriage, and education were the only ones associated with cervical cytology abnormalities. The overall agreement between the real-time PCR and Pap cytology screening methods was 78.96% (Kappa value of 0.12, 95% CI (0.00–0.243), *P* = 0.01).

**Conclusions:**

Non-16/18 HR HPV genotypes represented the largest proportion of HR HPV infections in this study. Women without cervical cytology abnormalities had the highest frequency of HR HPV infection. A large-scale community-based cohort study shall be designed and implemented to further identifying the persistent genotype and assessing the changes in cervical epithelial cell lines.

**Electronic supplementary material:**

The online version of this article (10.1186/s12885-019-5953-1) contains supplementary material, which is available to authorized users.

## Background

The World Health Organization estimates that nearly 530,000 women worldwide are diagnosed with cervical cancer every year and that 275,000 die from the disease. Cervical cancer is renowned as the third most common cause of cancer in women globally, of which almost 70% occurs in developing countries [1, 2]. In Ethiopia, the age-standardized incidence and mortality rates are estimated as 26.4 and 18.4 per 100,000, respectively, four- and ninefold higher that the incidence and mortality rates in Western Europe [1].

Cervical cancer has been recognized as an unusual outcome of a sexually transmitted infection, and the etiology is limited to a few *human papillomavirus* (HPV) genotypes. The association between HPV and cervical cancer is a universal fact, and variability among the different types is geographically limited. With optimal testing systems, HPV DNA can be identified in almost all specimens of invasive cervical cancer, and infection of the cervix with HPV is the main cause of cervical cancer [2]. One of the major reasons identified for the progression and development of cervical neoplasia among women who are repeatedly infected is ineffective cell-mediated immunity [3].

Of all HPV genotypes, more than 40 have been identified from anogenital mucosa samples and most are transmitted sexually. HPV genotypes 16, 18, 31, 33, 35, 39, 45, 51, 52, 56, 58, 59, 66 and 68 are classified as the high-risk (HR) group, which predicts cervical cancer [4]. The major phases in cervical oncogenesis include infection of the metaplastic epithelium of the cervical transformation zone with high-risk HPV infection, viral persistence and clonal progression of the persistently infected epithelium to cervical pre-cancer, and invasion [5].

In sub-Saharan Africa, HPV-associated cervical cancer is one of the major causes of morbidity and mortality. A lack of strong initiatives as well as sustainable cervical cancer prevention programs and services have been identified as potential causes of the high incidence rate in most countries [6]. In Eastern Africa, approximately 35.8% of women are estimated to harbor cervical HPV infection at any given time, and 76.5% of invasive cervical cancers are associated with HPV 16 or 18 [7]. Moreover, only 0.6% of the total female population aged 18–69 years in Ethiopia is screened every 3 years, representing 1.6% urban women and 0.4% rural women, which demonstrates that screening practice is underdeveloped and that the overall coverage is very limited [8, 9]. This study produced substantial information with relevant data regarding the burden of HR HPV infection and cervical cytology abnormalities in the intended setting.

## Methods

An institutional-based cross-sectional study design was employed at three selected obstetrics and gynecology clinics of Addis Ababa, Ethiopia, from June to October 2015. The study was conducted among women who visited the Family Guidance Association of Ethiopia Addis Ababa Area Reproductive Health Clinic, Hemen Maternal and Children Health Specialty Center, and SinamokshEthio Women’s Health Special Clinic. The study population consisted of women who visited the clinics for any gynecological purposes, including cervical cancer screening, and fulfilled the inclusion criteria. A nonprobability convenience sampling technique was used to select the study sites, considering the scope and volume of services provided. As these health facilities provide cervical cancer screening services and have a significant volume of client visits, they were potential sites for this study and among the very few sites providing this service consistently in the city. All women who visited each clinic during the study period and who were eligible for this study were consecutively added until the number of clients reached the calculated minimum sample size. A total of 366 women were enrolled in the study.

Sociodemographic characteristics, sexual behaviors and other risk-factor variable responses were gathered using a structured questionnaire (Additional file 1). HR HPV DNA and Pap screenings were performed following the standard operating procedure (Additional file 2 and 3). The cytological examination was performed by two pathologists whose degree of expertise was Medical Doctor with Diploma in Pathology and Cytology. Agreement between HR HPV and Pap smear results was assessed by Cohen’s Kappa coefficient by recoding the findings into two categories (Negative and Positive). The results were entered into EpiData software Version 13.0, and the data were analyzed using STATA Software Version 11.0. Descriptive statistics, proportions and the actual number of cases were used to describe frequency outputs for categorical variables and arithmetic means for the average age of the participants. Cross-tabulations were performed to explore and display relationships between two categorical variables. Chi-square statistics were employed to assess differences between two categorical variables. Multivariate logistic regression analysis (adjusted odds ratio) was applied to evaluate the strength of the association of the various potential risk factors with the presence of HR HPV infection and cervical cytology abnormalities. Positive and negative percentage agreement and overall percentage agreement were assessed for HR HPV DNA PCR and Pap smear screening methods. A *P*-value of less than 0.05 was considered statistically significant.

## Results

### Study subjects and sociodemographic characteristics

A total of 366 participants between 18 and 68 years of age were enrolled in this study. The mean age was 42.7 ± 10.7 SD. Most study subjects, 296/366 (80.9%), were within the range of 31–60 years. In terms of residence, 352 (96.2%) participants visited the study clinics from the Addis Ababa area. Of the total number of participants, 287 (78.4%) were married; among these, 71 (24.7%) was married for the first time before 18 years of age. Regarding parity, 29 (7.9%) of the participants had > 5 complete pregnancies and deliveries; 281 (76.8%) women were parity 1 to 5.

Participant employment status was also assessed, and 248 (67.8%) of the study participants were self-employed. Regarding educational status, the highest proportion comprised those with Diploma or Degree and above qualification (158/366; 43.4%), and only 39 (10.7%) were unable to read and write (Table 1).

**Table 1:** Sociodemographic characteristics of the study participants, Addis Ababa, Ethiopia, June to October 2015

Variable	Number	%
Age		
18–30	53	14.48
31–60	296	80.87
> 60	17	4.64
Residence		
Addis Ababa	352	96.2
Outside Addis Ababa	14	3.8
Marital Status		
Single	32	8.7
Married	287	78.4
Widowed	28	7.7
Divorced	19	5.2
Age at first marriage		
< 15	39	10.66
15–17	32	8.74
> =18	295	80.6
Parity		
0	56	15.3
1 to 5	281	76.8
> 5	29	7.9
Employment status		
Employed (Government/Private/NGO)	108	29.5
Self-employed	248	67.8
Unemployed	10	2.7
Education		
Unable to read and write	39	10.7
Elementary	64	17.5
High school	105	28.7
Diploma/Degree and above	158	43.2

### Burden of High-risk *Human papillomavirus* and its genotypes

**Fig. 1:**
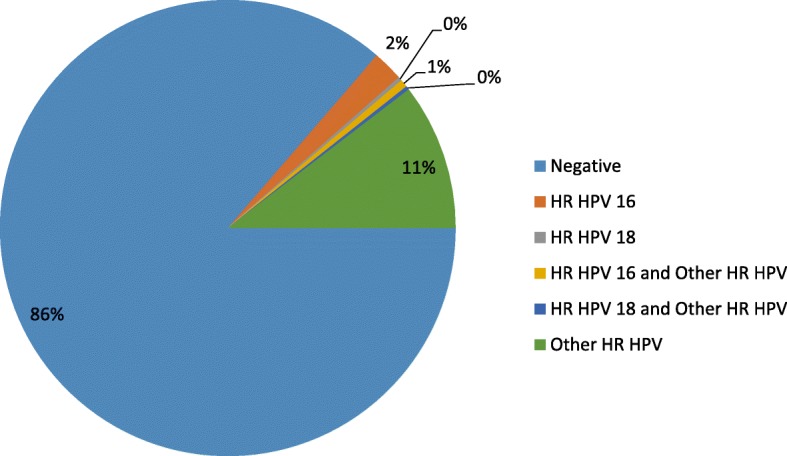
HR HPV genotype proportional distribution among all HR HPV-positive participants, Addis Ababa, Ethiopia, June to October 2015

The overall burden of HR HPV infection in this study was 50/366 (13.7%). Among the HR HPV-positive cases, 8 (16%) were identified as having HR HPV 16 genotype, 38 (76%) had “other HR HPV” (HR HPV genotypes 31, 33, 35, 39, 45, 51, 52, 56, 58, 59, 66, or 68), 2 (4%) had genotype 16 together with “other HR HPV” genotypes, 1 (2%) had genotype 18 together with “other HR HPV” genotypes, and 1 (2%) had genotype 18. The HR HPV genotype distribution showed that “other HR HPV” types dominated over genotypes 16 and 18 (Fig. 1).

Despite the low proportion, multiple infections were identified for HR HPV 16 and “other HR HPV” as well as for HR HPV 18 and “other HR HPV”, with proportions of 4 and 2%, respectively. The proportion of HR HPV-positive cases was 26, 72, and 2% in age ranges 18–30, 31–60, and > 60, respectively. Age range 31–60 was found to have the highest proportion of positivity (76, 95% CI (71.3–80.1%)), which was statistically significant.

The association between HR HPV infection with sociodemographic and reproductive health, sexual behavior, and other risk factors was analyzed through bivariate analysis using the chi-square test. Age (*P* = 0.000), parity (*P* = 0.017), age at first marriage (*P* = 0.027), education (*P* = 0.003), condom use during sexual intercourse (0.011), cigarette smoking (0.000), and family history of cervical cancer (0.003) were significantly associated with HR HPV infection. Ever use of any type of contraceptive, age at first sexual intercourse, more than one lifetime sexual partnership, history of STD, alcohol consumption, and HIV serostatus, with *P*-values of 0.106, 0.266, 0.334, 0.824, 0.227, and 0.688, were not significantly associated.

In multivariate analyses using logistic regression, only “other HR HPV” type was significantly associated with residence, employment status, and HIV serostatus, with P-values of 0.037, 0.01, and 0.041, respectively (Tables 2 and 3). Individuals who visited the clinics from outside Addis Ababa were 8.12 times more likely to have “other HR HPV” type infection than those who were from Addis Ababa. Furthermore, the likelihood of having “other HR HPV” infection among unemployed individuals was 9.2 times higher than for employed individuals. Compared to diploma or degree holders, women who were not able to read and write were less likely to be infected with “other HR HPV” types (Table 2).

**Table 2:** Association of “other HR HPV” genotypes with sociodemographic factors, Addis Ababa, Ethiopia, July to October 2015

Sociodemographic	Response Category	“other HR HPV” positivity	*COR(95% CI)	*P*-value	**AOR(95% CI)	*P*-value
Age	18–30	10 (26.32)	Ref^a^			
	31–60	27 (71.05)	0.43 (0.19,0.96))	0.038	0.6 (0.01,1.77.9)	0.36
	> 60	1 (2.63)	0.27 (0.03,2.27)	0.228	0.33 (0.02,4.9)	0.42
Residence	Addis Ababa	35 (70.0)	Ref^a^			
	Out of Addis Ababa	3 (6.0)	2.35 (0.62,8.81)	0.207	8.12 (1.14, 57.9)	0.037^b^
Marital status	Married	28 (56.0)	Ref^a^			
	Unmarried	7 (14.0)	2.795 (1.096,7.128)	0.031	3.22 (0.73, 14.29)	0.123
	Widowed	3 (6.0)	1.05 (0.299,3.716)	0.934	1.80 (0.40,8.24)	0.445
	Divorced	0	0			
Age at first marriage	< 15	2 (4.0)	Ref^a^			
	15–17	4 (8.0)	0.25 (0.025,2.59)	0.248	2.15 (0.17,0.48)	0.55
	> = 18	32 (64.0)	0.14 (0.029,0.646)	0.012	4.98 (0.47,52.2)	0.18
Parity	0	7 (14.0)	Ref^a^			
	1 to 5	28 (56.0)	0.72 (0.3,1.8)	0.471	4.15 (0.90,19.2))	0.067
	> 5	3 (6.0)	0.79 (0.2,3.3)	0.743	6.78 (0.66,72.4)	0.101
Employment status	Employed (Government/Private/NGO)	12 (24.0)	Ref^a^			
	Self employed	22 (44.0)	0.79 (0.37,1.66)	0.531	1.22 (0.548, 3.07)	0.67
	Unemployed	4 (8.0)	5.1 (1.26,20.73)	0.023	9.17 (1.6, 52.22)	0.01^b^
Education	Unable to read and write	1 (2.0)	Ref^a^			
	Elementary	6 (12.0)	3.75 (0.43,32.48)	0.23	4.86 (0.4,59.4)	0.215
	High school	11 (22.0)	4.33 (0.54,34.77)	0.168	12.39 (01.01151.12.5))	0.049
	Diploma/Degree and above	20 (40.0)	5.34 (0.69,41.21)	0.108	14.06 (1.12,176.6)	0.041^b^

^*^COR-Crude Odds Ratio, ^**^AOR-Adjusted Odds Ratio, ^a^Reference, ^b^There is a statistically significant association

**Table 3:** Association of “other HR HPV” genotypes with sexual behavior and other risk factor variables, Addis Ababa, Ethiopia, June to October 2015

Sexual behavior and other risk factor variables	Response Category	“other HR HPV” positivity	*COR(95% CI)	*P*-value	**AOR(95% CI)	*P*-value
Ever use of contraceptive	Yes	24 (48.0)	Ref^a^			
	No	14 (28.0)	0.01 (0.3,1.2)	0.164	0.62 (0.28,1.40)	0.251
Age at first sexual intercourse	< 15	3 (6.0)	Ref^a^			
	15–17	10 (20.0)	1.39 (0.35,5.53)	0.637	0.30 (0.03,2.62)	0.275
	> = 18	25 (50.0)	0.82 (0.23,2.9)	0.753	0.13 (0.01,1.04)	0.054
More than one lifetime partnership	Yes	25 (50.0)	Ref^a^			
	No	13 (26.0)	0.65 (0.32,1.31)	0.229	0.79 (0.34,1.86)	0.591
Condom use during sexual intercourse	Yes	14 (28.0)	Ref^a^			
	No	24 (48.0)	0.53 (0.3,0.9)	0.076	0.66 (0.27,1.66)	0.38
History of STD	Yes	6 (12.0)	Ref^a^			
	No	32 (64.0)	1.24 (0.5,3.1)	0.641	2.62 (0.7,9.8)	0.151
Cigarette smoking	Yes	2 (4.0)	Ref^a^			
	No	36 (72.0)	0.53 (0.1,2.6)	0.435	0.86 (0.13,5.6)	0.87
Family history of cervical cancer	Yes	4 (8.0)	Ref^a^			
	No	34 (68.0)	0.01 (0.2,1.9)	0.402	1.24 (0.3,5.)	0.76
Alcohol consumption	Usually	3 (6.0)	Ref^a^			
	Occasionally	16 (32.0)	1.32 (0.4,4.9)	0.68	1.33 (0.27,6.2)	0.72
	Never	19 (38.0)	0.75 (0.2,2.7)	0.67	0.8 (0.16,4.0)	0.79
HIV serostatus	Negative	25 (50.0)	Ref^a^			
	Positive	3 (6.0)	2.67 (0.7,10.3)	0.156	5.73 (1.06,30.9)	0.042^b^

^*^COR-Crude Odds Ratio, ^**^AOR-Adjusted Odds Ratio, ^a^Reference, ^b^There is a statistically significant association

### Abnormal cervical cytology

**Fig. 2:**
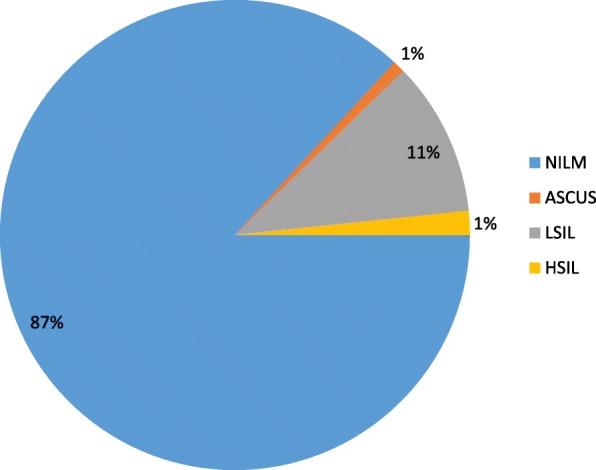
Proportion of Pap smear cytology results among the study population, Addis Ababa, Ethiopia, June to October 2015

Overall, Pap smear abnormalities were observed in 13.1% (48/366) of the study subjects. Among the abnormalities, 3 (6.3%), 39 (81.3%), and 6 (12.5%) were ASCUS, LSIL, and HSIL, respectively (Fig. 2). Among the abnormal cytology categories, LSIL abnormality showed the highest frequency. Low-grade squamous intraepithelial lesion (LSIL) and high-grade intraepithelial lesion (HSIL) rates in the 31–60 age category were 33 (84.62%) and 6 (66.67%), respectively, higher compared to the other age categories (Table 4). The association between age category and abnormal cytology was assessed by Fisher’s exact test and found not to be statistically significant (*P*-value = 0.180).

**Table 4:** Frequency of abnormal Pap smear cytology by age category, Addis Ababa, Ethiopia, June to October 2015

Abnormal Pap smear cytology	Age Categories (year)			*P*-value	Total
	18–30	31–60	> 60		
	No (%)	No (%)	No (%)		
ASCUS	0 (0.00)	3 (7.5)	0 (0.00)		3 (6.25)
LSIL	4 (10.26)	33 (84.62)	2 (5.13)	0.180	39 (81.3)
HSIL	0 (0.00)	4 (66.67)	2 (33.33)		6 (12.5)
Total	4 (8.33)	40 (83.33)	4 (8.33)		48 (100.0)

**Table 5:** Association of HSIL abnormal cytology with “age, age at first marriage and educational status”, Chi-square analysis, Addis Ababa, Ethiopia, June to October 2015

Variable		Frequency No (%)	Chi-square	*P*-Value
Age	18-30	0 (0)	11.84	0.003
	31-60	4 (66.67)		
	>60	2 (33.33)		
Age at first marriage	<15	1 (16.67)	10.99	0.004
	15-17	3 (50)		
	>=18	2 (33.33)		
Educational status	Illiterate	3 (50)	10.66	0.014
	Elementary	0 (0)		
	High School	1 (16.67)		
	Diploma/Degree and above	2 (33.33)		

In addition, a significant association with any of the risk factor variables was not observed for LSIL abnormalities according to the Chi-square test. In contrast, HSIL abnormal cytology was significantly associated with age, age at first marriage and educational status, with *P*-values of 0.003, 0.004 and 0.014, respectively (Table 5).

Ever use of any type of contraceptive, oral contraceptive use, age at first sexual intercourse, more than one lifetime sexual partnership, frequency of condom use, frequency of cigarette smoking, history of STD, and alcohol consumption were not significantly associated with abnormal Pap cytology (*P*-value > 0.05).

HR HPV genotypes were compared with cytological abnormalities, and the results are summarized in Table 6. The overall HR HPV genotype frequency among the total normal cytology results was 40/318 (12.6%). As shown in Tables 6, 40 (80%) of HR HPV-positive individuals had normal cytology (NILM); 5 (10%), 4 (8%), and 1 (2%) had LSIL, HSIL, and ASCUS, respectively. Among the total number of HR HPV-positive individuals, HR HPV 16 was found in two of the cases of HSIL abnormality, and the remaining two were caused by “other HR HPV” genotypes. HR HPV 18 was only found in NILM, but HR HPV 16 was identified both in NILM and HSIL. Nonetheless, “other HR HPV” genotypes were found across all stages (Table 6).

**Table 6:** HR HPV genotypes compared to Pap smear cytology findings, Addis Ababa, Ethiopia, June to October 2015

HR HPV Genotypes	NILM	ASCUS	LSIL	HSIL
HR HPV 16	6 (12%)	0 (0%)	0 (0%)	2 (4%)
HR HPV 18	1 (2%)	0 (0%)	0 (0%)	0 (0%)
Other HR HPV	30 (60%)	1 (2%)	5 (10%)	2 (4%)
HR HPV 16 and other HR HPV	2 (4%)	0 (0%)	0 (0%)	0 (0%)
HR HPV 18 and other HR HPV	1 (2%)	0 (0%)	0 (0%)	0 (0%)
Total	40 (100%)	1 (100%)	5 (100%)	4 (100%)

### Percent agreement between HR HPV DNA PCR and conventional pap smear cytology

Agreement between HR HPV DNA PCR and conventional Pap smear cervical cancer screening methods was analyzed using positive, negative, and overall percent agreement and the Kappa statistic. The positive and negative percent agreement was found to be 87.7 and 22.4%, respectively. However, the overall percent agreement was 79.0%, and the Kappa value was 0.12 (95% CI (0.00–0.24), *P*-value =0.01). The overall percent agreement findings reveal significant agreement between HR HPV DNA PCR and conventional Pap smear cytology screening methods (*P* < 0.05).

## Discussion

This study mainly aimed to assess the burden of HR HPV and cervical cytology abnormalities, along with potentially associated sociodemographic, sexual behavior, and reproductive health variables, in three Obstetrics and Gynecology and reproductive health clinics in Addis Ababa, Ethiopia. In this study, the overall HR HPV burden was 13.7%. “Other HR HPV” genotypes (31, 33, 35, 39, 45, 51, 52, 56, 58, 59, 66, or 68 types) were the most frequent (76%) genotypes identified in this study, followed by HR HPV 16 (16%). The overall prevalence of abnormal cytology was also 13.1%. Approximately three-fourth (72%) of the HR HPV-infected women were in the age range of 31 to 60 years, and this was significantly associated with abnormal cytology. HR HPV was found in 12.6% of normal cytology reports. Moreover, residence, occupation, and HIV serostatus were significantly associated with HR HPV infection.

In this study, the overall HR HPV burden was 13.7%, a finding that was consistent with previous studies reported from different parts of Ethiopia [14, 15], at 13.2 and 15.8%, respectively. In contrast, our finding was much lower than those in two other studies from Ethiopia [16, 17]. This difference might be because the participants in the first study [16] were women with cervical complaints and all samples were cases of cervical dysplasia, which may result in higher values. In our study, however, cytological samples were obtained from women who did not necessarily have cervical dysplasia or cervical complaints. Similarly, the difference from the other report [17] may be due to the study site chosen, as that study was conducted in the only specialized cancer center in Ethiopia, which would increase the probability of observing a large number of positive HR HPV cases. In addition, approximately 34% of those study participants were HIV positive, which might also have contributed to the higher rate of HR HPV [12]. Our finding was lower than the estimated prevalence reported for all HPV genotypes (high-risk and low-risk types) from sub-Saharan African countries (21.8%) [11] and Nigeria (21.6%) [12].

To date, studies conducted in Ethiopia [14, 16] have reported that HR HPV 16 is the predominant type. In contrast, the most frequent genotypes identified in the present study were “other HR HPV” genotypes (31, 33, 35, 39, 45, 51, 52, 56, 58, 59, 66, or 68 types), contributing 76%, followed by HR HPV 16 (16%). Our finding is comparable with that in a worldwide meta-analysis review [10], which reported that the predominant genotype in Eastern Africa was HR HPV 52, followed by HPV 16. Another study [18] found that HPV-positive women in sub-Saharan Africa were less likely to be infected by HPV 16 than were women in Europe. Similarly, another study [19] examining paraffin-embedded cervical tissues reported that HPV 52 (25.5%) and 58 (22%) were the most frequent genotypes. This difference in genotype frequency in various studies might be due to geographic variation and host immunogenetic factors. Regardless, HPV 16 appears to be less influenced by immune status than other HPV genotypes. This fact, coupled with impairment in cellular immunity, may contribute to the presence of HPV genotypes other than HPV 16 in some populations [19].

Multiple HR HPV type infections were found in 7.9% of HR HPV-positive individuals in a study by Mohammed et al. in Northeastern Nigeria [20], which is comparable to the findings of the present study (6%). In contrast, the 17.5% of multiple infections in a study conducted on Ethiopian and Sudanese women [19] was relatively higher than that in the present study. This may be due to the nature of the samples processed in that study [19], which included tissue blocks with cervical intraepithelial neoplasia or carcinoma, and the possibility of infection by more than one type of HR HPV may increase in such cases [16].

An age-specific HPV infection study in South Africa [13] reported that the highest frequency (74.6%) of infections was found in women older than 25 years. Similarly, another study from Addis Ababa, Ethiopia, reported that 50.6% of HR HPV-infected women were in the age range of 30–50 years [17]. These studies are consistent with our finding that 72% of the HR HPV-infected women were 31–60 years of age. However, the significant association between age group in the bivariate analysis (*P* < 0.05) was not significant in the multivariate analysis. This is similar to the results of the study conducted in Gurage Zone, Ethiopia [14]. In contrast, a study by Andall B in Trinidad (33) showed that the highest (63%) prevalence of HPV infection was observed among women aged < 30 years (*P* < 0.0001), with a peak in the age range of 21 to 25 years. This might be due to the detection of low-risk HPV in addition to HR HPV.

In this study, residence, occupation, and HIV serostatus were significantly associated with HR HPV infection in multivariate analysis. This finding was comparable with a study [24] reporting that occupation and residence are significantly associated with HPV infection. Nonetheless, the study by Muluken et al. in Tikur Anbessa Specialized Hospital, Addis Ababa, Ethiopia [17], reported that HIV and residence were not significantly associated with HR HPV prevalence. This might be due to differences in sampling, type of participants, and data collection methods.

In our study, ever use of any type of contraceptive, age at first sexual intercourse, and more than one lifetime sexual partnership were not associated with HR HPV infection. This outcome is comparable to the findings of Mega AC et al. in rural Nigeria [21].

The overall abnormal cytology burden in the present study was 13.1%, which was lower than that in a similar study from Ethiopia [17, 22] and another from South Africa [13]. This difference might be due to the presence of a large number of HIV-infected individuals, who are not easily able to resolve infection and experience progression to the development of precancerous to cancerous lesions [23]. In our bivariate analysis, age at first marriage and educational level were significantly associated with HSIL Pap smear abnormality (*p*-value 0.004 and 0.014), consistent with a study reported by Abel et al. [22].

Furthermore, our study presents high-risk HPV genotypes with cervical cytology findings. HR HPV 16 was found in 50% of HSIL reports, and “other HR HPV genotypes” were the most frequent finding for LSIL. Similarly, for women who had normal cervical cytology results, the most frequent genotypes were “other HR HPV” genotypes. According to the meta-analysis by Gary C. et al. [18], the most common HR HPV type in HSIL among women with and without cervical neoplastic diseases was HR HPV 16, which was consistent with our findings. In contrast to the same study [18], which reported HR HPV 16 as the predominant genotype in LSIL and NILM, all the LSIL and NILM results in our study were attributed to “other HR HPV” genotypes. Moreover, “other HR HPV” genotypes were observed across all grade levels of cytological findings. As reported in various studies, HPV-positive women in sub-Saharan Africa are less likely to be infected with HR HPV 16 than are their counterparts in Europe ([18–20], and). Interestingly, the present study also revealed that 12.6% of women with NILM were positive or any type of HR-HPV infection. This is comparable to a study [25] from West Africa reporting that 13% of women with normal cytology results were positive for HR HPV. In such situations, the women may continue to have an increased risk of HSIL during the interval between the first and next screening [26].

## Conclusions

The burdens of HR HPV infection and cervical cytology abnormalities presented in this study are consistent with the few previous local studies and reviews in Ethiopia but somehow lower than the estimated prevalence for sub-Saharan Africa. Unlike previous studies, “other high-risk HPV” genotypes contributed considerably to the overall HR HPV burden. Multiple-type infections were found in sexually active women. The highest frequency of HR HPV positivity was in women without cervical cytology abnormalities. Hence, the interval between the primary and secondary HPV screening for HR HPV positives and negatives needs to be defined separately. The performance of the Abbott Real-Time HR HPV DNA PCR and Pap smear cytology screening methods may need to be further evaluated against histologically confirmed results. In addition, the screening program for early-age sexually active women should be further promoted in various health settings. The Ministry of Health should also further consider the possibility of introducing vaccines targeting other oncogenic HPV types in addition to genotypes 16 and 18. A large-scale community-based cohort study shall also be designed and implemented to determine the national burden and the molecular epidemiology of persistent HR HPV types and cervical cytology abnormalities which will help to recommend the ideal screening algorithm considering the local context. This will significantly contribute to the national preventive public health strategies against cervical cancer.

## Additional files

Additional file 1: Questionnaire. (DOCX 20 kb)

Additional file 2: HR HPV detection procedure using the Abbott Real-Time PCR method. (DOCX 17 kb)

Additional file 3: Abnormal cytology diagnosis procedure using the conventional Pap smear method. (DOCX 17 kb)

## Data Availability

The datasets used and/or analyzed during the current study are available from the corresponding author on reasonable request.

## Abbreviations

ASCUS: Atypical squamous cells of undetermined significance
HIV: Human immunodeficiency virus
HR HPV: High-risk human papillomavirus
HSIL: High-grade squamous intraepithelial lesion
LSIL: Low-grade squamous intraepithelial lesion
NILM: Negative for intra-epithelial lesions and malignancy
PCR: Polymerase chain reaction
STD: Sexually transmitted disease

## References

[1] Shaniqua L, Jeanne M (2014). Update on prevention and screening of cervical cancer. Baishideng publishing group Inc. World J ClinOncol.

[2] Cervical cancer action. Progress in cervical cancer prevention; the CCA report. 2012.

[3] Xavier FB, You-Lin Q, and Xavier C. The epidemiology of human papillomavirus infection and its association with cervical cancer. Int J Gynecol Obstet. Elsevier Ireland Ltd 2006;94:8–21.

[4] Center of Disease control and prevention. Epidemiology and prevention of vaccine-preventable diseases. 2015.

[5] Adeola F, Manga M (2013). Utilization of human papillomavirus (HPV) DNA detection for cervical cancer screening in developing countries: a myth or reality. Afr J Micro Research.

[6] Hugo De V, Laia A, Charles L, Caria JC, Vikrant S, Cecily B, et al. The burden of Human Papilloma Virus infections and related diseases in sub-Saharan Africa. NIH Public Health Access. 2013;31(05):32–46.

[7] ICO Information center on HPV and Cancer-Ethiopia. Human Papilloma virus and related cancers. Fact sheet.2014.

[8] Federal Democratic Republic of Ethiopia Ministry of Health. Guideline for cervical cancer prevention and control in Ethiopia. 2015.

[9] Farhad A, Rainer K, Belaynew W (2012). Understanding cervical cancer in the context of developing countries. Ann Trop Med Public Health.

[10] Silvia de S, Mieria D, Xavier C, Gary C, Laia B, Nubia M (2007). World-wide prevalence and genotype distribution of cervical human papilloma virus DNA in women with normal cytology: a meta-analysis. Lancet Infect Dis.

[11] Karly S, Silvia de S, Philippe M (2009). Epidemiology and prevention of human papillomavirus and cervical cancer in sub-Saharan Africa: a comprehensive review. Trop Med Int Health.

[12] Nweke GI, Banjo AA, Abdulkareem FB, Nwadike UV (2013). Prevalence of human papilloma virus DNA in HIV positive women in Lagos University teaching hospital (LUTH) Lagos, Nigeria. Br J Micro Res.

[13] Richter Karin, Becker P, Horton A, Dreyer G (2013). Age-specific prevalence of cervical human papillomavirus infection and cytological abnormalities in women in Gauteng Province. South African Medical Journal.

[14] Sami-Ramzi LB, Christof P, Mauritis NC, Hartmut G, Ralph JL (2014). Cervical human papilloma virus prevalence and genotype distribution among hybrid capture 2 positive women 15 to 64 years of age in the Gurage zone, rural Ethiopia. Infect Agents Cancer..

[15] Ruland R, Prugger C, Schiffer R, Regidor M, Lellé RJ (2006). Prevalence of human papilloma virus infection in women in rural Ethiopia Attat hospital. Eur J Epidemiol.

[16] Bekele A, Baay M, Mekonnen Z, Suleman S, Chatterjee S (2010). Human papillomavirus type distribution among women with cervical pathology – a study over 4 years at Jimma hospital, Southwest Ethiopia. Tropical Med Int Health.

[17] Muluken D, Solomon G, Yirgue G, Dawit W, Bekure T, Wondwossen E (2010). Human papilloma virus infection and genotype distribution in relation to cervical cytology abnormalities and HIV-1 infection at TikurAnbessa teaching hospital.

[18] Gary C, Silvia F, Mieria D, Nubia M, Luisa LV. HPV-type distribution in women with and without cervical neoplastic diseases. Science Direct. 2006:S3/26–34.

[19] Ebba A, Abrham A, Muntasir EH, Ibrahim EH, Laerence Y, Wude M (2013). Genotyping of human papilloma virus in paraffin embedded cervical tissue samples from women in Ethiopia and Sudan. J Med Virol.

[20] Mohammed MM, Adeola F, Yusuf MA, Aliyu UE, Danladi BA, Hamidu UP (2015). Epidemiological patterns of cervical human papilloma virus infection among women presenting for cervical cancer screening in North-Eastern Nigeria. Infectious Agents Cancer.

[21] Megan AC, Julia CG, Kayode OA, Nicolas AW, Akinfolarin CA, Sholom W, et al. A population-based cross-sectional study of age-specific risk factors for high risk human papillomavirus prevalence in rural Nigeria. Infect Agents Cancer. 2011;6(12):1–8. 10.1186/1750-9378-6-12 PMC3162906 21801395

[22] Abel G, Ayalew A, Gizachew A (2013). The prevalence of pre-cancerous cervical cancer lesion among HIV-infected women in southern Ethiopia: a cross sectional study. PLoS One.

[23] Laia B, Mireia D, Xavier C, Elena F, Xavier F (2010). And Selviade’S. Cervical human papilloma virus prevalence in 5 continents: meta-analysis of 1 million women with normal cytological findings. J Infect Dis.

[24] Quamrun N, Farhana S, Andil A, Jessica YI, Mustafizur R, Fatema K (2014). Genital human papilloma virus infection among women in Bangladish. PLoS One.

[25] Long Fu XI, Papa T, Cathy W, Stephen E, Birama D (2003). Prevalence of specific types of human papillomavirus and cervical squamous intraepithelial lesions in consecutive, previously unscreened, west-African women over 35 years of age. Int J Cancer.

[26] Nicole JP, Nienke JV, Danielle AM, Peter JF, Chris JL (2017). HPV positive women with normal cytology remain at increased risk of CIN3 after a negative repeat HPV test. Br J Cancer.

