# Supplementary material for: Burden and genotype distribution of high-risk Human Papillomavirus infection and cervical cytology abnormalities at selected obstetrics and gynecology clinics of Addis Ababa, Ethiopia
Source: BMC Cancer. 2019 Aug 5;19:768. doi: 10.1186/s12885-019-5953-1 (PMC6683490; doi:10.1186/s12885-019-5953-1)
Supplement: Supplementary file 1 — Questionnaire. (DOCX 20 kb) [file 12885_2019_5953_MOESM1_ESM.docx]

| **QUESTIONAIRE** | | | | | | | | | | | |
| --- | --- | --- | --- | --- | --- | --- | --- | --- | --- | --- | --- |
| Name of Health Facility: | | | | | | | | | | | |
| Participant Code**፡- KHPV/SIN /000** | | | | | | | | | Date**:** | **Time፡-** | |
| Please make a#**√**; mark in the provided boxes. | | | | | | | | | | | |
| **SNo.** | | **Variable** | **Response** | | | | | | | | **Remark** |
| **Part I-Socio-demographic characteristics** | | | | | | | | | | | |
| 1 | | Age: | ________Years | | | | | | | |  |
| 2 | | Address: |  | | | | | Addis Ababa | | |  |
|  |  |  |  | | | | | Outside Addis Ababa | | |  |
| 5 | | Marital Status |  | | | | Married | | | |  |
|  |  |  |  | | | | Unmarried | | | |  |
|  |  |  |  | | | | Widowed | | | |  |
|  |  |  |  | | | | Divorced | | | |  |
| 6 | | Age at first marriage |  | | | | <15 years of age | | | |  |
|  |  |  |  | | | | 15-17 years of age | | | |  |
|  |  |  |  | | | | >=18 years of age | | | |  |
| 7 | | Parity: |  | | | | 0 | | | |  |
|  |  |  |  | | | | 1-5 | | | |  |
|  |  |  |  | | | | >5 | | | |  |
| 8 | | Occupation: |  | | | | Employed**(**GO/Private/NGO) | | | |  |
|  |  |  |  | | | | Self-employed | | | |  |
|  |  |  |  | | | | Unemployed | | | |  |
| 10 | | Educational Status: |  | | | | Unable to read and write | | | |  |
|  |  |  |  | | | | Elementary/Junior(1-8) | | | |  |
|  |  |  |  | | | | High School(9-12) | | | |  |
|  |  |  |  | | | | Diploma/Degree and Above | | | |  |
| **Part-II Reproductive and Sexual Health risk factors characteristics** | | | | | | | | | | | |
| 1 | Previous history of contraceptive use | | |  | | Yes | | | | |  |
|  |  |  |  |  | | No | | | | |  |
| 2 | If “Yes” for question # 1, which type of contraceptive you used to have? | | |  | | Oral Contraceptive (OCP) | | | | |  |
|  |  |  |  |  | | IUCD | | | | |  |
|  |  |  |  |  | | Implant | | | | |  |
|  |  |  |  |  | | Depo (Injectable) | | | | |  |
|  |  |  |  |  | | Permanent (Tuba ligation) | | | | |  |
|  |  |  |  | Other, specify: | | | | | | |  |
| 3 | If you used to have OCP, for how many year/s you stayed? | | |  | | <5 years | | | | |  |
|  |  |  |  |  | | 5 years | | | | |  |
|  |  |  |  |  | | >5 years | | | | |  |
| 4 | Age at first sexual inter-course; | | |  | | <15 | | | | |  |
|  |  |  |  |  | | 15-17 | | | | |  |
|  |  |  |  |  | | >=18 | | | | |  |
| 5 | More than one life time sexual partnership | | |  | | Yes | | | | |  |
|  |  |  |  |  | | No | | | | |  |
| 6 | History of condom use during sexual intercourse | | |  | | Some times | | | | |  |
|  |  |  |  |  | | Always | | | | |  |
|  |  |  |  |  | | No | | | | |  |
| 7 | Do you smoke cigarette | | |  | Some times | | | | | |  |
|  |  |  |  |  | Always | | | | | |  |
|  |  |  |  |  | No | | | | | |  |
| 8 | Do you have previous history of sexually transmitted disease | | |  | Yes | | | | | |  |
|  |  |  |  |  | No | | | | | |  |
| 9 | Do you have previous family history of cervical cancer | | |  | Yes | | | | | |  |
|  |  |  |  |  | No | | | | | |  |
| 10 | Do you consume alcohol | | |  | Usually | | | | | |  |
|  |  |  |  |  | Occasionally | | | | | |  |
|  |  |  |  |  | Never | | | | | |  |
| 11 | HIV Sero-status | | |  | Negative | | | | | | From client’s medical record |
|  |  |  |  |  | Positive | | | | | |  |
