# Supplementary material for: Burden and genotype distribution of high-risk Human Papillomavirus infection and cervical cytology abnormalities at selected obstetrics and gynecology clinics of Addis Ababa, Ethiopia
Source: BMC Cancer. 2019 Aug 5;19:768. doi: 10.1186/s12885-019-5953-1 (PMC6683490; doi:10.1186/s12885-019-5953-1)
Supplement: Supplementary file 2 — HR HPV detection procedure using the Abbott Real-Time PCR method. (DOCX 17 kb) [file 12885_2019_5953_MOESM2_ESM.docx]

# HR HPV detection procedure using Abbott RealTime PCR method

**Intended use**

The Abbott RealTime High Risk HPV is a qualitative in-vitro test for the detection of DNA from 14 high Risk Human Papilloma virus (HR HPV) genotypes 16, 18, 31, 33, 35, 39, 45, 51, 52, 56, 58, 59, 66, and 68 in clinical specimens.

**Principle**

A primer mix consisting of three forward primers and two reverse primers targeting a conserved L1 region is used to amplify HPV targets. Signal for fourteen HR HPV genotypes (HPV 16, 18, 31, 33, 35, 39, 45, 51, 52, 56, 58, 59, 66, and 68) is generated with the use of fluorescent labeled probes. Internal Control (IC) amplicons are generated with a primer set targeting an endogenous human beta globin sequence and is detected with the IC specific probe. The Abbott RealTi*m*e HR HPV assay detects the endogenous human beta globin sequence as sample validity control for cell adequacy, sample extraction and amplification efficiency. Probes for HPV 16, HPV 18, non-HPV 16/18 genotypes (Other HR HPV) and IC are labeled with different fluorophores allowing their signals to be distinguishable in a single reaction.

**Procedure for using the m2000sp instrument**

1. Thaw control- and amplification reagents. In mean time:
2. Vortex each specimen for 15-20 seconds. Liquid has to be on bottom of vial. Specimens collected with the Abbott Cervi-Collect tubes, can be loaded without cap directly on the m2000sp.
3. When controls are thawed. Vortex each control 15-20 seconds. Ensure that contents are on bottom.
4. Place the controls and the patient specimens into the m2000sp sample rack. Bar codes on tube labels must face right for scanning.
5. Open the mSample preparation system_DNA_ reagent pack(s). Prepare the mWash2_DNA_ by adding 70mL of Ethanol (95% - 100%; do not use denaturants!) to the mWash2_DNA_ bottle. Mix by gently inverting and check if there are no crystals.
6. Vigorously mix the mMicroparticles_DNA_ and pour into the 200mL reagent vessel.
7. Initiate the sample extraction protocol.
8. While the m2000sp is performing the sample preparation, switch on and initialize the m2000rt for a 15-minute warm-up.
9. Load the amplification reagents and the master mix tube on the m2000sp worktable.
10. Initiate the m2000sp master mix addition protocol.
11. After the m2000sp has completed the addition of samples and amplification reagents, seal the reaction plate.
12. In PCR-3 room, place the reaction plate in the m2000rt and initiate the RealTime HR HPV assay protocol.
13. After the m2000rt has completed the amplification and detection protocol, remove the reaction plate and dispose.
14. Clean and decontaminate all working areas.

**RESULT INTERPRETATION**

| **Result** | **Reporting** |
| --- | --- |
| HPV 16 | HPV 16 is detected but  HPV 18 and other HR HPV are not detected. |
| HPV 18 | HPV 18 is detected but  HPV 16 and other HR HPV are not detected. |
| Other HR HPV | Other HR HPV is detected but  HPV 16 and HPV 18 are not detected. |
| HPV 16, Other HR HPV | HPV 16 and Other HR HPV are detected  HPV 18 is not detected. |
| HPV 18, Other HR HPV | HPV 18 and Other HR HPV are detected  HPV 16 is not detected. |
| HPV 16, HPV 18 | HPV 16 and HPV 18 are detected  Other HR HPV is not detected. |
| HPV 16, HPV 18, and Other HR HPV | HPV 16 and HPV 18 and Other HR HPV are detected |
| Not Detected | HR HPV is not detected* |

*****“*Not detected” does not mean that the patient is “Negative” for HPV infection. The assay should be interpreted in conjunction with other clinical and laboratory findings.*

**QUALITY CONTROL**

**INTERNAL**

Detection of inhibition and/or Cell Inadequacy:

The HR HPV assay detects the endogenous human beta globin sequence as Internal Control (IC) signal to evaluate cell adequacy, sample extraction and amplification efficiency.

Negative and Positive control

The controls need to be processed together with the samples prior to running the amplification portion of the assay.

The negative control is formulated with DNA containing IC sequence. The only signal detected for negative control should be the IC signal in the Cy5 channel.

The positive control is formulated with DNA containing HPV 16 (VIC), HPV 18 (NED), HPV 58 (FAM) and IC (Cy5) sequences.
