# Supplementary material for: Burden and genotype distribution of high-risk Human Papillomavirus infection and cervical cytology abnormalities at selected obstetrics and gynecology clinics of Addis Ababa, Ethiopia
Source: BMC Cancer. 2019 Aug 5;19:768. doi: 10.1186/s12885-019-5953-1 (PMC6683490; doi:10.1186/s12885-019-5953-1)
Supplement: Supplementary file 3 — Abnormal cytology diagnosis procedure using the conventional Pap smear method. (DOCX 17 kb) [file 12885_2019_5953_MOESM3_ESM.docx]

# Abnormal cytology diagnosis using conventional Pap smear method

**Principle**

Pap test is a test done on cervical cells scarped from the surface as well as the exfoliated cells in order to look for morphological abnormalities that indicate premalignant conditions. The cells are smeared on a slide, fixed with ethanol and stained for visualization under a microscope.

**Specimen**

The optimal time for gynecologic cytology specimen collection is two weeks after the start of the patient’s last menstrual period. She should avoid vaginal medications, spermicides, and douches for 48 hours and inter course for 24 hours before the day of her appointment.

**Procedure**

1. Complete out the Pap smear requisition completely.
2. Label the frosted end of the slide with the patient’s name and date of birth in pencil, date and time of collection.
3. Do not remove the glass slide from the paper holder for safety and convenience.
4. The speculum is lubricated with warm water only, not lubricant jelly.
5. It is positioned to expose the cervix at the end of the speculum.
6. If large quantities of mucus or exudates are present, gently remove by patting with dry gauze without disturbing the epithelium.
7. Three specimen sources can be sampled: endocervix, ectocervix, and vaginal pool.
8. Endocervical specimens are collected with a cytobrush using a 360 degree rotation within the canal (see diagram).
9. Ectocervical specimens are collected with a spatula using a 360 degree rotation just inside the cervix, and sampling both the transformation zone and ectocervix (see diagram I).
10. Vaginal pool specimens are collected by obtaining a drop of vaginal fluid from the posterior fornix using the posterior lip of the speculum.
11. Endocervical and ectocervical specimens are smeared onto the glass slide *immediately* after collection and *immediately* fixed with cytology fixative (see diagram). Best results are obtained with a uniform thin smear.
12. If a vaginal pool specimen is collected in addition to ectocervical and/or endocervical specimens, collect the vaginal pool specimen first, place one inch from the end of the glass slide and do not smear initially.(Please refer to diagram I.)
13. Next collect the remaining specimens and mix the sample with the lower portion of the vaginal pool specimen.
14. Smear the specimens thinly and evenly across the slide and fix *immediately* with cytology fixative.
15. Fold the card board container with patient’s name and date of birth and date and time of collection with a ball point pen.
16. Deliver the sample and test requisition to the laboratory.
17. Pap smears can also be picked up by the courier on a schedule.

**Quality Control**

Specimen adequacy will be assessed using Bethesda 2001 system. Bethesda 2001 designates specimen adequacy as “satisfactory” or “unsatisfactory.” Specimen quality indicators such as the presence or absence of a transformation zone component, or of obscuring inflammation or blood, are reported after the adequacy designation. The criteria for the classification of adequacy as “satisfactory” or “unsatisfactory” are based on:

1. Cellular material covers at least 10 percent of the area under the coverslip (125mm^2^) at a “normal” or “usual” cell density, or that 10 percent or 125mm^2^ of the slide is actually covered or obscured because of the presence of cells.
2. Smear contains between 8,000 and 12,000 well-preserved, well-visualized cells. This assessment is not done by counting cells manually. Images below depicting low-power (~4×) microscope fields with a low number of cells should be used as a comparison for asequacy.
